# Dietary advice for muscularity, leanness and weight control in *Men’s Health* magazine: a content analysis

**DOI:** 10.1186/1471-2458-14-1062

**Published:** 2014-10-11

**Authors:** Toni M Cook, Jean M Russell, Margo E Barker

**Affiliations:** Human Nutrition Unit, Department of Oncology, School of Medicine, University of Sheffield, Beech Hill Road, Sheffield, S10 2RX UK; Corporate Information and Computing Services, University of Sheffield, 10-12 Brunswick Street, Sheffield, S10 2FN UK

**Keywords:** Magazines, Masculinity, Body image, Diet, Qualitative research

## Abstract

**Background:**

The dietary content of advice in men’s lifestyle magazines has not been closely scrutinised.

**Methods:**

We carried out an analysis of such content in all 2009 issues (n = 11) of *Men’s Health* (*MH*) focusing on muscularity, leanness and weight control.

**Results:**

Promotion of a mesomorphic body image underpinned advice to affect muscle building and control weight. Diet advice was underpinned by a strong pseudo-scientific discourse, with citation of expert sources widely used to legitimise the information. Frequently multiple dietary components were advocated within one article e.g. fat, omega-3 fatty acids, thiamine, zinc and high-glycaemic index foods. Furthermore advice would cover numerous nutritional effects, e.g. strengthening bones, reducing stress and boosting testosterone, with little contextualisation. The emphasis on attainment of a mesomorphic body image permitted promotion of slimming diets.

Advice to increase calorie and protein intake to augment muscle mass was frequent (183 and 262 references, respectively). Such an anabolic diet was advised in various ways, including consumption of traditional protein foods (217 references) and sports foods (107 references), thereby replicating muscle magazines’ support for nutritional supplements. Although advice to increase consumption of red meat was common (52 references), fish and non-flesh sources of protein (eggs, nuts & pulses, and soy products) together exceeded red meat in number of recommendations (206 references). Advice widely asserted micronutrients and phytochemicals from plant food (161 references) as being important in muscle building. This emphasis diverges from stereotypical gender-based food consumption patterns.

Dietary advice for control of body weight largely replicated that of muscularity, with strong endorsement to consume fruits and vegetables (59 references), diets rich in nuts and pulses and fish (66 references), as well as specific micronutrients and phytochemicals (62 references). Notably there was emphasis on fat-burning, good fats and consumption of single foods, with relatively little mention of dietary restriction.

**Conclusions:**

Despite the widespread use of scientific information to endorse dietary advice, the content, format and scientific basis of dietary content of *MH* leaves much to be desired. The dietary advice as provided may not be conducive to public health.

## Background

The print media has an acknowledged role as a communicator of food and nutrition-centred advice and information [1]. Magazines, therefore, affect consumer knowledge of food, nutrition and health and influence food choices through both reflecting and possibly creating acceptable social norms associated with food consumption and health [2–4].

There has been much research attention as to the nature and construction of information about food, dieting, nutrition and health in women’s magazines [3, 5–7]. In contrast, although it has been observed that contemporary lifestyle magazines for men include such topics as cooking, health and well-being [8] there has been little coherent study of these food and health messages. An early analysis [9] comparing the content of women’s and men’s magazines reported that both contained high levels of health information, however men’s magazines had more articles focusing on diet and exercise and cited expert opinion more frequently.

There has been substantial research interest in men’s lifestyle magazines from a sociological perspective [8, 10–12]. The traditional masculine gender ideal, often referred to as hegemonic masculinity [13], whereby men are dominant, aggressive and unemotional i.e. the stereotypical ‘man’s man’, was seen to prevail in all forms of media directed at men. However, contemporary ideals of masculinity have evolved towards a more unisex lifestyle whereby men are encouraged to explore their feminine sides, including concern about appearance and health. In keeping, the media has responded by creating magazines that not only address traditional masculine ideals of power, money and physical fitness, but also advise on all manners of consumerism, health and wellbeing [14].

Where diet advice in men’s magazines has been considered, this has been in very general terms. Ricciardelli quantified the overall level of dietary advice in men’s lifestyle magazines in relation to ideals of muscularity [12]. Stibbe focused on the rhetoric used to promote (and often overstate) men’s needs for meat, convenience food and beer in *Men’s Health* (*MH*) content [14]. Neither study examined the specifics or quality of the diet information provided.

In 2009 *MH* was reported to be the world’s largest men’s magazine, sold in over 39 countries [15]. It has a current UK print readership of 1,041,000, which is above all other well-known men’s publication such as *FHM*, *Men’s Fitness*, *GQ* and *Esquire. MH* is the fifth best-selling general monthly magazine. The majority of its audience is men between 15-34 years, from a middle class demographic, with good spending power [16]. This study aims to examine the format and specifics of the dietary advice in *MH* in relation to muscularity & leanness and weight control.

## Methods

### *Men’s Health*magazine

We sampled one complete set of issues (n = 11) for the year 2009. All articles with reference to diet (food, nutrients and eating patterns) were identified and entered into NVivo software (NVivo9, QSR, 2009) for analysis.

#### Analytic process

Initial attempts at coding food and nutrition messages following published schemes [17] were unsuccessful because of the complexity and specificity of the diet messages. Therefore a grounded theory approach was used to develop a manageable taxonomy [18]. The aim was to examine representations of nutrition and food messages within *MH*. This was achieved by applying detailed, systematic, exhaustive coding of all articles dealing with diet. We initially coded according to three broad themes: 1) Diet Information (specific foods, nutrients and food components, consumption patterns) 2) Format of Diet Information and 3) Fitness and Health Outcomes.

Each article was subject to multiple coding, serving to summarise its primary focus whilst allowing for the identification of detail [19, 20] i.e. an article would be coded for each individual food or nutrient mentioned and the health and fitness context(s) in which the diet claim was made; as well as format of diet information. A total of 86% of the diet advice pages had 10+ codes. One author carried out this process. Over 3000 different codes were recorded at the initial stage of coding.

**Figure 1:**
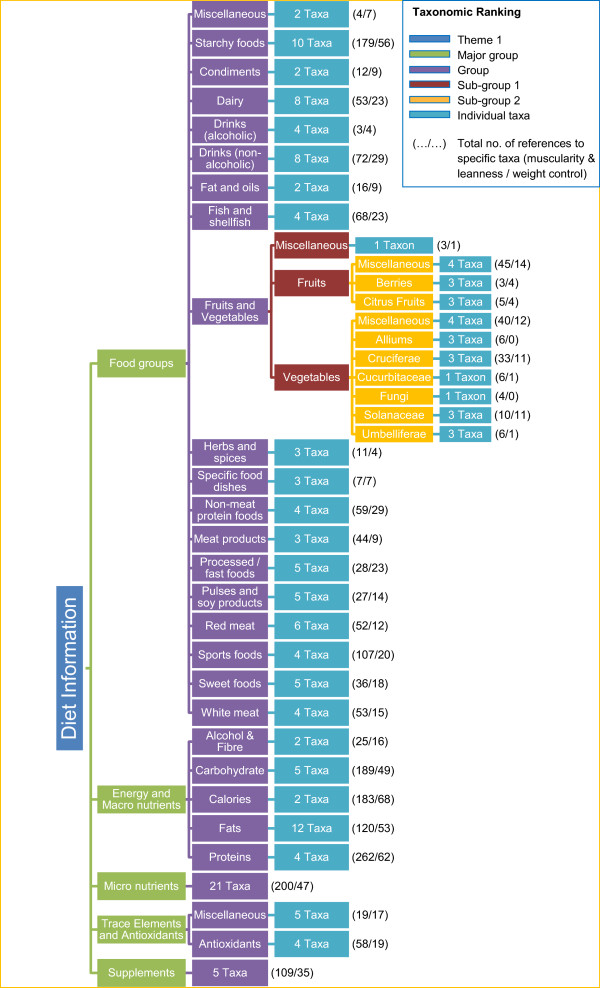
**Coding scheme for dietary content of Theme 1.** The coding scheme used to classify the foods, nutrients and food components of dietary content in *Men’s Health*, presented as a taxonomy of 5 ranks. Individual taxa in the final rank are not named. The first number provided refers to the number of references in that taxa in relation to M&L, while the second number is the number of references in that taxa in relation to weight control. (As not all taxa are shown in the figure some data within the Results section are not present in the figure).

**Figure 2:**
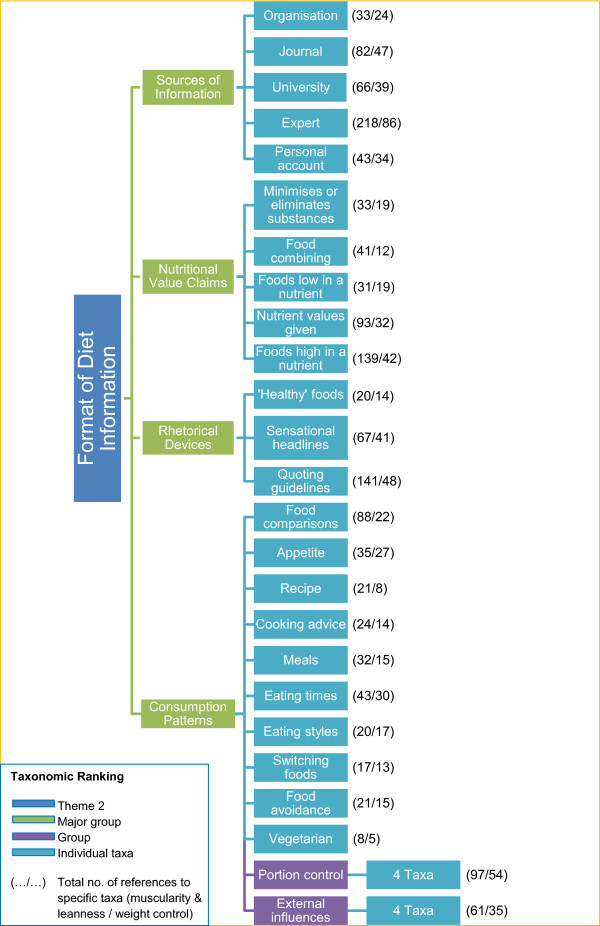
**Coding scheme for the format of diet information of Theme 2.** The coding scheme used to classify the format of the diet information in content of *Men’s Health*, presented as a taxonomy of 4 ranks. Individual taxa in the final rank are not named. The first number provided refers to the number of references in that taxa in relation to M&L, while the second number is the number of references in that taxa in relation to weight control. (As not all taxa are shown in the figure some data within the Results section are not present in the figure).

**Figure 3:**
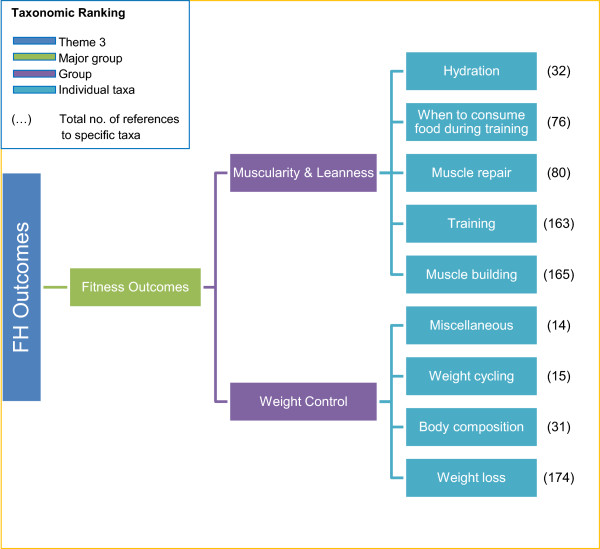
**Coding scheme for fitness outcomes of Theme 3.** The coding scheme used to classify fitness outcomes in content of *Men’s Health*, presented as taxonomy of 3 ranks. Individual taxa are included.

Further reflection in conjunction with the other members of the research team allowed progression from codes to categories in line with principles of grounded theory [19]. This involved a protracted process whereby categories were refined and joint decisions made over handling of similar categories. The final coding scheme was developed by consensus involving all three authors.Figure 1 shows the coding scheme for the foods, nutrients and food components of Theme 1. The scheme is presented as taxonomy. The final rank of taxa has not been labelled in the figure because of the sheer number of these. The number of references per taxa relate specifically to the fitness outcomes of muscularity & leanness and weight control.Figure 2 shows the coding scheme used to categorise the format of diet information. Initial groupings were into four broad categories: sources of information, nutritional value claims, rhetorical devices and consumption patterns. Further differentiation was into 24 taxa, of which two were further subdivided into four taxa. The number of references per taxa provided in the figure relate specifically to the fitness outcomes of muscularity & leanness (M&L) and weight control.The coding scheme for the fitness outcomes of Theme 3 is given in Figure 3. These fitness outcomes were split into two broad categories of muscularity & leanness and weight control, and further differentiated into 9 individual taxa.

In this analysis we explore relationships between fitness outcomes of Theme 3 and dietary messages of Theme 1 and 2. We used the matrix query function in NVivo to quantify relationships. Associations were verified by crosschecking with original articles.

## Results

### Frequency of advice

The metrics for frequency of dietary advice in the magazines are given in Table 1*.* Content was heavily weighted towards dietary advice for muscularity & leanness ends, with lesser emphasis on body weight control. In total, diet advice comprised 7.5% of total content.

**Table 1:** **Frequency of dietary advice in relation to muscularity & leanness and weight control in the sample of** ***MH*** **magazines**

No of issues	11
Total number of pages	2165
Number of references	750
Median number of advice pages in an issue	35
No of references to:	
Muscularity and Leanness	516
Weight control	234
Dietary advice	162 pages (7% of content)

### The format of advice

There were at least two dedicated diet features per issue: *Personal Trainer*, which advised on diet in relation to a prescribed work-out, and *Core Nutrition* which focused on nutritional advice for muscle building. The authors of Personal Trainer and Core Nutrition were *MH*’s editors and fitness experts*,* with input from a series of experts ranging from dietitians to sports nutritionists to naturopaths; the formal qualifications of the authors were not provided. An additional section was introduced midway through 2009 entitled *Nutrition-Refuel*, which centred on building a strong body through nutrition. As part of these regular features a piece entitled *Snack-Off* compared the nutritional profiles of two snacks, one recommended for muscle-building and the other for leanness (fat-burning). Advice for muscularity & leanness and weight control often featured together. Such positioning was exemplified by a double page article, which was standard within the *Core Nutrition* feature, featuring recipe and nutritional information for two versions of the same meal. One version made claims for bulking-up, whilst the other made claims for weight loss. Both meals featured the same core foods of meat and vegetables with fewer (or no) carbohydrate- and fat-rich foods in the slimming down version:

*On the side you’ll ditch standard chips or spuds in favour of sweet potatoes. These are higher in carbs than ordinary spuds but they also pack more quality as well as quantity because their low GI means they provide a steady supply of energy, rather than a spike in blood sugar levels. This helps your body to use the steak’s protein to reconstruct muscle tissue more effectively.*

(*MH*, July 2009: 73)

Articles varied in length (from one brief sentence up to 6 pages), but typically comprised a single page, including the regular features of *Nutrition-Refuel, Personal Training,* and *Core Nutrition*. Although articles differed in length, the vast majority contained external endorsement. A spectrum of sources was used to endorse claims: independent experts (304 references) such as sports nutritionists and general practitioners; published journal papers (129 references); University research results and academic staff (105 references), and independent organisations (57 references). Other forms of endorsement were fewer; sometimes a *MH* cover model or celebrity detailed his recommended diet and exercise regimen for achievement of a mesomorphic physique (accompanied by images of the end result), whilst in other instances readers’ testimonials of the success of a transformative dietary and exercise regimen were the focus (77 references). Before-and-after photographs sometimes accompanied these. In two issues there was a dietary article by an alpha-female, specifically a young actress and fitness expert, on how to achieve a svelte and lean body with accompanying by-line and photograph(s) of the scantily clothed author.

Quoting academic nutrition specialists was common:

*Iron is vital for getting oxygen to your red blood cells, which then carry it to your muscles, staving off fatigue. “And the vitamin C in lemon makes the iron in veg much easier for your body to absorb”, says Sanders. It actually converts plant-based iron into a form similar to that found in fish and red meat. So adding a squeeze of citrus to your greens gives them the benefits of a sirloin or tuna steak for a fraction of the cost. Sanders recommends getting your vitamin C from lemons or limes, strawberries, tomatoes, peppers, and broccoli, and your plant-based iron from kale, leeks and spinach*.

(*MH*, August 2009: 51)

A popular form of communication was a compendium of short snippets of dietary advice in headline form abstracted from the conclusions of various and disparate published studies:

*Get a milk moustache for more muscle. A study in the American Journal of Clinical Nutrition…*

*Suck an espresso for express strength. A study in the Journal of Strength and Conditioning Research*…

(*MH*, October 2009: 141)

Many of the headline nutrition statements were derived from a single published study (exceptionally the citation was a systematic review), with import of a minute subset of results, which were invariably turned into simple and unconditional dietary directives to the reader. Little or no context was given as to whether the research was preliminary, whether there was other supporting evidence, and whether the study findings could be applied to men in general. Rarely were qualifications made, and even when made were dismissed:

*A study in Medicine & Science in Sports and Exercise found that rats given an extract of apple experienced a 10% increase in strength, a 30% drop in body fat and significantly less muscle fatigue. You may not think you have much in common with the furry vermin, but due to the high polyphenol concentration found in apples, you’ll enjoy the same benefits as they did.*

(*MH*, May 2009: 145)

Men’s need for food was reduced to a mechanical and functional requirement, specifically to invoke a physiological effect. Dietary and nutritional advice was specific, precise and imperative - detailed meal plans and strictures as to quantities and timing of consumption were common (54 and 31 references for muscularity & leanness and weight control, respectively). The nutritional effects were portrayed as instant, unconditional and absolutely certain.

The authority of nutritional science was strongly emphasised. Indeed science was attributed with powers beyond the natural, rationale and mundane. Consumption of the correct foods and nutrients in specific quantities would result in transformative and magical effects on the body through the portal of nutritional science:

*Nutrition science transforms a classic pie into a fat-stripping, muscle-building miracle*

(*MH*, Jan/Feb 2009: 71)

*Studies at the University of Rovira i Virgili in Spain found the most significant health improvements came from eating the right combination of different nuts each day: three walnuts, eight hazelnuts and eight almonds to be precise. As well as improving cholesterol and blood pressure and delivering a cocktail of brain-training omega-3s, bone-strengthening calcium and antioxidant vitamin E to oil your vital equipment downstairs, they found this magic blend - weighing in at 40 g - significantly reduced belly fat.*

(*MH*, October 2009: 69)

Whilst the focus of this analysis was muscularity & leanness and weight control, articles rarely provided information on these topics alone. Frequently there would be coverage of numerous nutritional effects, with little explanation of context or scientific background, save for mention of an expert or published paper. For example, in one page of a two-page spread focusing on high protein foods for weight control entitled *The World’s Best Protein Foods* (*MH*, May 2009: 141) there was also reference to 18 other dietary components (*What to eat with it* [what to eat with the protein food]): bread, pasta, potato, condiments, green tea, avocado, pizza, snack foods, fibre, carbohydrate, high-Glycaemic Index (GI) foods, low GI foods, bad cholesterol, fat, fatty acids, omega-3 fatty acids, saturated fat, iron, magnesium, selenium, vitamin B12, B3, thiamine, zinc, trace elements, tryptophan, antioxidants, supplements. These were recommended for a spectrum of physiological and health effects: recovery after exercise, reduction of the amount of mercury absorbed from fish, lowering of blood sugar, minimising free radical damage, absorption of weight loss aiding-fat, inhibition of the release of mucus when breathing, nervous and digestive health, bad cholesterol, sleep and feeling good, elasticity of arteries, reducing arterial stiffness and blood sugar spikes. This set of recommendations seemed to be trawled from nutritional science journals – the foods recommended to source the array of nutrients and to accompany each protein food were culinary disjoint, for example green tea with tuna, pinto beans with pork tenderloin, pizza with walnuts.

### Specific advice for muscularity & leanness

Advice to increase intake of calorie and protein (including recommendations for individual amino acids) to build muscle was frequent (183 and 262 references, respectively).

*Follow in the footsteps of the weights room juggernauts: competitive body builders. They employ a monstrous 55:15:30 carbohydrate-protein-fat ratio to gain mass. You should also eat at least 500-1,000 calories more than you need a day (the average man should consume around 2,550 calories per day). This will flood your system with an overload of nutrients that are begging to be turned into muscle….* (*MH*, October 2009: 141)

*Follow James’ typical day of eating to give your muscles everything they need.****Breakfast 4 egg whites and 1 yolk scrambled, wholemeal toast, 1/2 grapefruit.****Protein to give your metabolism a kick-start, low-GI carbs for energy and vitamin C for fat-burning…****Mid-morning Snack Whey protein shake, approx 40 g nuts and dried fruit mix.****Keeping your protein intake high is vital*….. (*MH* October 2009: 92)

The foods recommended for increasing protein intake were various. Consumption of red meat was frequently recommended (52 references):

*“My nutritional philosophy is simple - eat meat….I’ll also eat a joint of beef wrapped in a herb jacket for a testosterone boost*…”

(*MH*, May 2009: 36)

Fish and non-flesh sources of protein (eggs, nuts & pulses, and soy products) superseded red meat in number of recommendations (68 and 138 references, respectively). These foods were not only recommended for protein content:

*Salmon, tuna and mackerel are rich in Coenzyme Q10 (which gives you energy), vitamin A and zinc (for testosterone) and protein (for muscle). Eat a serving per day.*

(*MH*, April 2009: 137)

Micronutrients such as vitamin A, zinc, iron, selenium and vitamin B12 were widely recommended to aid muscle building and muscle repair (200 references). Explicit endorsement of plant foods was common (161 references):

***“Radishes ….Why?****Their anti-inflammatory properties aid muscle recovery…”* (*MH*, March 2009: 120)

There was particular emphasis on the effects of a range of antioxidants and phytochemicals (77 references). Advice extended to claims that phytochemicals could affect sex hormones:

*Cabbage. Full of phyto-chemical IC3. A study at Rockefeller University, US also found when men were given doses of IC3, their girly oestrogen levels dropped while testosterone surged.*

(*MH*, April 2009: 137)

Traditional anti-oxidant micronutrients such as carotenoids and vitamin C were advocated:

*Get a couple of easy-peelers down your neck before training and you could be setting yourself up for bigger muscle gains at the end. Research carried out at the Linus Pauling Institute in Oregon, US found that the antioxidant properties of vitamin C enhances the production of nitric oxide, which dilates your blood vessels to enable vital nutrients to reach your muscles quickly post-training.*

(*MH*, May 2009: 145)

Dietary fat was both vilified and advocated for attainment of muscularity:

*Fuelling the transformation – Sports nutritionist Jane Griffin explains how James’ low-fat, seven-meal diet will help you achieve your body goals*

(*MH*, August 2009: 81)

*Eat fat* [natural fats as opposed to trans fats] *to burn fat*

(*MH*, July 2009: 145)

*Pan-roasted Lobster. Good for muscle building and great for easing stress (there’s killing involved) in just 20 minutes. Lobster is almost as high in protein as steak; but with less fat…*

(*MH*, June 2009: 94)

Advice to consume “good fats” was frequent (63 references), particularly to produce fat-burning of abdominal fat. Supplementation with omega-3 fatty acids and fish oils were frequently advised in this context. Mention of restriction of dietary fat as a method to improve leanness was slightly less common (57 references).

All manner of dietary supplements (109 references) and sports foods (107 references) were recommended for muscle augmentation and resistance training. The main categories endorsed were sports drinks, protein shakes, milk proteins, sports bars, multivitamin and mineral supplements, creatine, and CoQ10:

*Meal #7 – 10.00 pm – SCI-MX GRS-5 protein system shake and 2 tbsp of peanut butter. Peanut butter contains “good” fat, protein and fibre and the amino acid tryptophan – used to make mood-regulating serotonin. Peanuts have one of the lowest GIs of all nuts.*

(*MH*, August 2009: 81)

*Whey protein. Whey is rich in proteins vital to the manufacture of testosterone, your brawn hormone. Bracket your workouts with whey shakes*.

(*MH*, April 2009: 137)

### Advice for weight control

The food and nutrient advice around leanness for control of body weight largely replicated that of muscularity, with strong endorsement to consume fruits and vegetables (59 references), eggs, nuts, pulses & soy products (43 references) and fish (23 references). There was also endorsement of antioxidants and phytochemicals (36 references) and micronutrients (47 references) for weight loss:

***“Fresh Herbs (basil, coriander, mint)***

***How much?****2 g roughly chopped*

***Why?****They are high in vitamin K, calcium and iron – all of which are crucial in producing the glycogen for a healthy liver, which can help with weight loss. What’s not to like?”* (*MH*, March 2009: 120)

Although energy and calories were frequently mentioned in the context of weight control (68 references), it was in very general terms, almost paying lip service to the notion of energy balance, calorie counting or food restriction. In fact on occasions calories were dismissed in favour of restriction of carbohydrate:

*Count carbs not calories – “I aim to eat carbohydrates that are high in fibre and low in starch”, says Daniel. “Vegetables such as broccoli, spinach, Brussels sprouts and peppers are all low in calories so I can basically eat as much as I like. For refined carbs, I go for the minimally processed variety: raw rather than roasted nuts, and whole grains such as barley, quinoa and oats.” ….*

(*MH*, July 2009: 145)

Controlling portion size as a method of slimming was mentioned relatively infrequently (14 references), whilst specific diet plans providing set menus were advised more often (31 references). Similarly, as for leanness & muscularity outcomes, there was much advice to alter fat intake (53 references) with particular support for the consumption of omega-3 fats to burn fat (18 references).

## Discussion

Men’s lifestyle magazines have been implicated in promoting concern about body size through their emphasis on the muscular ideal [10–12, 21]. This emphasis was reflected in our data, with 7.5% of content devoted to diet advice for bulking-up, strength training and body weight control, exceeding that (4.1%) reported for 2004-2006 issues of *MH* [12].

Central to post-modern construction of hegemonic masculinity is the muscular male body; it signifies courage, power, strength, sex appeal and control [22–24]. Appearance and physical fitness ideals underpin aspirations towards muscularity [14, 25], while pursuit of health may also drive attainment of a lean physique. Empirical data suggest that men conceptualise health as fitness, with fitness embracing both physical fitness and being fit to cope with the gendered roles of everyday life [24]. *MH* content reinforces these perceptions by portraying a mesomorphic body shape as integral to masculinity, and embedding physical fitness and body shape within a health framework.

The prominence of weight loss advice in magazine content concurs with Riccardelli’s observations in men’s lifestyle magazines [12], but is at odds with contemporary Irish and British newspapers’ representation of men, food and body weight [26, 27]. Unbridled food consumption has been associated with manliness in studies investigating how food consumption can convey gender-role messages within Western societies [28, 29]; similarly British men participating in a slimming programme perceived dietary self-control and following slimming diets to be women’s domain [30]. Changes in gender relations (in affluent societies) has resulted in an acceptance that men can consume “healthy” “feminine” foods [31], and can be figure-conscious [32]. These social changes were reflected in *MH’s* diet advice.

The invocation of experts has been identified as intrinsic to the biomedical-scientific discourse in women’s magazines [33, 34], and has previously been noted to be rife in men’s lifestyle magazines [9, 35]. The external expert is represented as integral to reducing disease risk and optimising the body’s performance; the body is in effect construed as being controlled from the outside through the agency of nutritional or sports science. The conjunction between science and masculinity is recognised in the sociological literature around the cultural reproduction of gender [36, 37]; science is founded on being dispassionate, objective and unemotional, which are masculine ideals. A semiotic analysis of advertising and editorial content of men’s fitness magazines [35] concluded that emphasis on nutritional science epitomises seriousness, reason and control, reflecting the values of hegemonic masculinity.

Contextual detail as to who researched, funded and published cited articles should be an important element of translation of science to the popular arena, helping the reader to judge the credibility of the dietary message [38, 39]. The funding source was never provided in *MH*. While citation of external expert and publication source in *MH* content served to legitimise the catalogue of dietary exhortation, such citations were often “semi-attached figures” [40] only partially relevant to the advice. Journal citation was used in essence as “scientific branding” to give quality assurance to the dietary exhortation. Such “branding” is dubious because although scientific publication in a peer-reviewed journal means that the science meets a reasonable standard, it does not mean that a study’s findings are definite [41]. The practice of giving dietary advice on the basis of single study is not recommended [41].

Furthermore, the common format of communicating a series of nutritional information with diverse detail of mechanism and outcome was overwhelming. Improving men’s understanding of nutritional health and the nutritional value of food through provision of such information seems unlikely, given that an exceptionally high level of health literacy would be necessary to interpret and assimilate such multifaceted advice.

The popularisation of nutritional science research often involves inferring a strong degree of certainty as to the effects of diet [41, 42], and this process was clearly visible in *MH*’s advice. Indeed in some cases the dietary exhortations claimed magical effects on physiology and health. While Fores has noted that 20^th^ Century Western culture imbues science with totemic qualities [43], *MH* content did this explicitly.

*MH* advised high-calorie, protein-rich food and protein-based sports supplements within a training regimen for muscularity. A calorie- and protein-rich diet combined with resistance exercise is recognised to augment muscle mass [44–48]. In body-building sports, protein supplementation has been a longstanding feature of training and may have benefits [44, 45, 49, 50]. However, such emphasis has been questioned, as positive energy balance may be more important than dietary protein; protein guidelines for recreational athletes are similar to the general population [48].

Fish and plant food were widely advocated as protein sources, conflicting with Stibbe’s report [14] that meat-eating was overwhelmingly advised in *MH*, and incongruent with the literature that consumption of meat, particularly red meat, is a potent symbol of dominant masculinity [51–55]. Recommendations for non-meat foods challenge the accepted dietary mores of a patriarchal society - women are expected to consume the lighter meats, fish and vegetables [55, 56], foods considered too ‘feminine’ for men. Use of masculine metaphors and promise of improved sexual performance served to defeminise plant food and reinforce hegemonic masculinity. The use of masculine metaphors has previously been noted in *MH* articles [8].

Recommendations for consumption of plant food also stemmed from emergent scientific literature showing that anti-inflammatory compounds (various phytochemicals) in fruit and vegetables can reduce muscle damage after exercise [57–62]. *MH* content actively championed these for muscle recovery after training, augmentation of muscle and limiting body fat. However, recent studies have questioned the performance benefits of polyphenol supplementation [63, 64].

Omega-3 fatty acids and fish oil were also commonly advised for leanness ends, although current scientific opinion for such an effect is divided [65, 66]. This was juxtaposed with advice to restrict fat in line with general recommendations for body-building [67].

Diet supplements and sports foods were widely endorsed. Intense marketing and promotion of such supplements has been documented in body-building and sports magazines [44] even though scientific evidence to support many such claims is lacking [68, 69]. Although protein shakes and creatine have been shown to be useful supplements to increase muscle mass, the effectiveness of amino acids, ginseng, bicarbonate and multivitamin and mineral supplements in aiding muscle accrual or muscle recovery following resistance training has been disputed, and such supplements are not advised by National Sports Bodies [44]. Burke surveyed four body-building magazines and noted that supplements advertised and endorsed extended beyond supplements to increase muscle mass – there was heavy marketing of a range of products claiming effects that allow athletes to train harder, recover faster, reduce muscle injury and increase sexual function [44]. It seems that *MH* is replicating muscle magazines’ promotion of commercial sports supplements.

A recent analysis of men’s correspondence about weight loss as part of an on-line weight loss programme reported that that men dismissed conventional diet plans as a feminine method of losing weight – exercise accompanied by systemic alterations to dietary habits was preferred [30]. Surprisingly, *MH* advice featured a substantial number of diet plans alongside personal stories of weight loss experiences. In keeping with contemporary official weight loss recommendations [70], a low-fat diet was recommended alongside consumption of fruit and vegetables. Exhortations to consume plant food are contrary to normative gender-based consumption patterns [71, 72], and challenge the food ideals of hegemonic masculinity.

*MH* portrayed losing weight as easy - a similar stance has been observed in advertising messages in young women’s magazines [6]. In this vein there was much commendation of individual “super-foods”, for example broccoli, capsicums and avocados, which were portrayed as the magic bullets of weight loss. Similarly, omega-3 fats were widely advised as a quick dietary fix for fat-burning, albeit that scientific evidence for such an effect in humans is limited [65, 66]. Notably there was minimum emphasis on dietary energy restriction through portion size control or calorie-counting, even though restriction of dietary energy is central to body weight loss [70, 73, 74].

Interpretation of these data must countenance the limitations of the sampling procedure used, although an entire year’s issues of *MH* was sampled, we have examined only one magazine. Nevertheless *MH* is large in the genre of lifestyle/health magazines, having both a considerable on-line and print readership.

Because of the array of diet and nutritional messages relating to leanness and muscularity the process of categorisation of diet advice was challenging, not least when one short piece conveyed multiple dietary messages covering many outcomes. Therefore, interpretation of the absolute numbers of references in relation to the specifics of the advice should be cautious – it is possible that there has been an underestimate of dietary messages due to the sheer density and seemingly random nature of advice within an article.

The boundary between muscularity and leanness and weight loss was blurred because of the level of overlap, intertwining and convergence of these topics. In some instances it was clear that bulking-up was the desired outcome, but in other instances muscle development converged with advice on leanness, fat-burning and general training needs.

The coding scheme was developed specifically for this research and does not correspond to coding schemes used elsewhere thereby limiting comparison with studies of women’s magazines. There was no published coding scheme for men’s magazines probably due to the density and complexity of dietary information.

## Conclusion

In conclusion, it is overwhelming clear that magazine content glorified the alpha-male and endorsed hegemonic masculinity through its emphasis on the desirability of muscularity & leanness. This desirability was embedded within a scientific and biomedical discourse, which reinforced hegemonic masculinity through its emphasis on reason, control and factual imperiousness.

While the dietary advice to achieve the ends of leanness & muscularity reinforced the dominant food ideology that meat, particularly red meat, is associated with maleness, the scientific discourse gave license for consumption of feminine plant foods and promotion of weight loss diets. In essence the employment of the scientific discourse in the writing permitted promotion of a comprehensive diet. This promotion of plant foods and endorsement of slimming is congruent with public health policy for prevention of chronic disease.

However, the poor reporting of dietary information is not in the interests of public health, and may negate any public health benefit. Firstly, the sheer ubiquity of nutritional information, often relayed in technical terms, was overwhelming. It has been suggested that the language used in health messages should be simplified to consider differing levels of health literacy [75]. Importantly *MH* advice was often based on single scientific studies. The cherry picking of scientific evidence and simplistic translation of limited evidence into dietary imperatives is a flagrant misuse of scientific information. Further work to consider how actual readers of *MH* make use of this medley of information is needed. Furthermore, while advice to consume anabolic diets and use plant foods to repair muscle may have application for power athletes, its applicability for readers of *MH* is debatable.
